# Mitochondrial Heme Synthesis Enzymes as Therapeutic Targets in Vascular Diseases

**DOI:** 10.3389/fphar.2020.01015

**Published:** 2020-07-15

**Authors:** Trupti Shetty, Timothy W. Corson

**Affiliations:** ^1^Department of Ophthalmology, Eugene and Marilyn Glick Eye Institute, Indiana University School of Medicine, Indianapolis, IN, United States; ^2^Department of Pharmacology and Toxicology, Indiana University School of Medicine, Indianapolis, IN, United States; ^3^Department of Biochemistry and Molecular Biology, Indiana University School of Medicine, Indianapolis, IN, United States

**Keywords:** age-related macular degeneration, diabetic retinopathy, angiogenesis, neovascularization, ferrochelatase, electron transport chain, endothelial nitric oxide synthase, heme synthesis

## Introduction: Synthesis and Functions of Heme

Mitochondrial function in endothelial cells (EC) is interconnected by a mesh of signaling molecules that cross pathways often (Kluge et al., 2013). One such versatile biomolecule is heme. Heme is important for respiration, curbing oxidative stress, drug metabolism, and oxygen transport (Dailey and Meissner, 2013). The heme synthesis pathway and intermediates have been studied in detail over decades, with crystal structures and cloned genes available (Poulos, 2014). Intriguingly, heme is an important prosthetic moiety of key proteins of EC (Chiabrando et al., 2014b).

**Figure 1:**
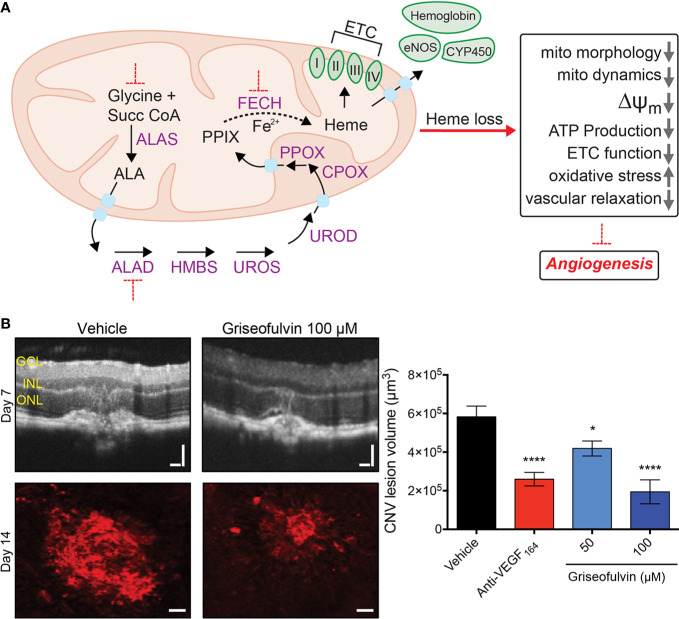
Schematic diagram of the heme synthesis pathway in the mitochondrion and effect of *Fech* inhibition *in vivo*. **(A)** Eight sequential steps in the heme synthesis pathway are depicted, along with some heme-containing proteins. Red dotted lines indicate blockade. **(B)** *Fech* inhibition using griseofulvin in the laser-induced choroidal neovascularization (CNV) mouse model. CNV was confirmed by optical coherence tomography (OCT). Griseofulvin treated eyes had significantly smaller neovascular lesions as seen in red agglutinin staining for vasculature. Retinal layers indicated: GCL, ganglion cell layer; INL, inner nuclear layer, ONL, outer nuclear layer; Scale bars for OCT images and agglutinin immunostaining are 100 and 50 µm, respectively. *p = 0.015; ****p = 0.0001 versus vehicle, ANOVA with Dunnett’s *post hoc* tests (n = 11–13 eyes per group). Anti-VEGF_164_ is a positive control antibody therapy. Figure modified from Basavarajappa et al., 2017 © 2017 The Authors, CC BY 4.0. Succ CoA, succinyl-CoA, ALA, 5-aminolevulinic acid; ALAS, ALA synthase; ALAD, ALA dehydratase; HMBS, hydroxymethylbilane synthase; UROS, uroporphyrinogen synthase; UROD, uroporphyrinogen decarboxylase; CPOX, coproporphyrinogen oxidase; PPOX, protoporphyrinogen oxidase; FECH, ferrochelatase; PPIX, protoporphyrin IX; eNOS, endothelial nitric oxide synthase; CYP450, cytochrome P450; ETC, electron transport chain; ΔΨ_m_ = mitochondrial membrane potential; mito, mitochondria.

In mammalian cells, heme synthesis is accomplished in the mitochondria and cytosol over a series of eight enzymatic reactions, followed by modification of heme in a couple of sub-hemylation steps (Nilsson et al., 2009; Hamza and Dailey, 2012; Dailey et al., 2017). Heme biosynthesis in cells other than erythrocytes is initiated by the rate-limiting enzyme aminolevulinic acid synthase (ALAS1) that catalyzes formation of 5-aminolevulinic acid (ALA) from succinyl-CoA and glycine (**Figure 1A**). ALA is exported into the cytosol and converted *via* several intermediates into coproporphyrinogen-III (CPO) by coproporphyrinogen oxidase (CPOX); CPO is then transported back into the mitochondria for the last two steps of the pathway. In the final step, ferrochelatase (FECH) incorporates ferrous iron into protoporphyrin IX (PPIX), synthesizing protoheme. Heme is then available to enable cellular processes by combining with enzyme subunits as a prosthetic group. For example, heme-iron is part of the catalytically active form of endothelial nitric oxide synthase (eNOS) (Raman et al., 1998). Similarly, different forms of heme are incorporated into mitochondrial respiratory complexes I–IV of the electron transport chain (ETC) (Kim et al., 2012). Of course, the majority of heme is used for incorporation into hemoglobin during erythropoiesis (Korolnek and Hamza, 2014) and some (primarily in the liver) for the synthesis of cytochrome P450s, responsible for xenobiotic metabolism (Correia et al., 2011).

Apart from being a prosthetic cofactor for enzymes, heme’s regulated production ensures that active iron is sequestered before it can promote formation of reactive oxygen species (ROS) (Ryter and Tyrrell, 2000). Hence, heme plays a crucial role in ROS homeostasis in the mitochondria, without which many mitochondrial processes would be damaged (Alonso et al., 2003). One key regulator involved in detoxification of ROS and stimulating mitochondrial biogenesis is proliferator-activated receptor gamma coactivator 1α (PGC1α) (Austin and St-Pierre, 2012). PGC1α regulates ALAS1 expression in the liver, linking heme synthesis directly to the nutritional state of cells (Handschin et al., 2005). Fasting-induced PGC1α was found to be essential for vascular growth and pathological angiogenesis (Saint-Geniez et al., 2013). Here, we review recent studies that have identified an unexpected link between angiogenesis and heme synthesis, offering exciting therapeutic relevance to vascular diseases like retinopathy of prematurity (ROP), proliferative diabetic retinopathy (PDR), and wet age-related macular degeneration (AMD).

## Heme Synthesis Proteins as Angiogenesis Mediators

The terminal heme synthesis enzyme, ferrochelatase, encoded by *FECH*, was the first heme pathway component to be identified as a druggable target in pathological angiogenesis. FECH blockade (both genetically and pharmacologically) reduced proliferation, migration and endothelial tube formation in microvascular ECs. This effect was specific to ECs; FECH inhibition had a negligible effect on non-endothelial ocular cell proliferation. This anti-angiogenic effect was also seen *in vivo*: mice with a partial loss-of-function *Fech*^m1Pas^ point mutation formed reduced neovascular lesions in the eye in the laser-induced choroidal neovascularization (L-CNV) model with features of wet AMD, as did mice with ocular *Fech* knockdown or inhibition (**Figure 1B**). In addition, FECH was overexpressed in and around these lesions, and in human wet age-related macular degeneration eyes (Basavarajappa et al., 2017). Moreover, FECH was upregulated, particularly in neovascular tufts, in the oxygen-induced retinopathy (OIR) mouse model of ROP (Pran Babu et al., 2020). The mechanisms of how heme contributes to EC physiology and drives angiogenesis are now beginning to be understood.

## Mechanisms of Heme Regulation of Angiogenesis

### Mitochondrial Function

Inhibition of heme synthesis has varying impact on the hemoproteins of the ETC (Vijayasarathy et al., 1999; Atamna et al., 2001). Heme *b* and *c* are present in complexes II and III, whereas complex IV has two groups of heme *a*, made after two consecutive modifications to protoheme (Kim et al., 2012). We recently showed that loss of heme *via* blockade of the terminal enzyme FECH in retinal ECs specifically causes complex IV dysfunction with negligible effects on other complexes of the ETC (Shetty et al., 2020). Complex IV protein and activity were significantly decreased by small molecule or genetic inhibition of FECH, but partially restored after heme supplementation. This loss in complex IV was accompanied by a depolarized mitochondrial membrane. Furthermore, heme depletion damaged both oxidative phosphorylation and glycolysis in retinal and choroidal ECs, along with a decrease in mitochondrial fusion and elevated ROS. This work characterized the direct effect of heme blockade on EC metabolism for the first time (Shetty et al., 2020).

Another recent study elucidated the contribution of the serine synthesis pathway to heme and EC metabolism (Vandekeere et al., 2018). Inhibition of the serine synthesis enzyme phosphoglycerate dehydrogenase (PHGDH) reduced glycine (substrate for the first step of the heme synthesis pathway), leading to an indirect decrease of heme enzymes and an eventual reduction in heme production in ECs. This also caused mitochondrial defects like reduced respiration, smaller mitochondria, increased fission, reduced fusion, and elevated mitophagy. Neonatal mice with silenced PHGDH had reduced retinal vascularization and reduced vessel area in the brain, heart, and kidney. Additionally, another group demonstrated that complex III is essential for EC proliferation (but not migration) in macrovascular ECs. Conditional knockout of EC-specific complex III led to reduced retinal, lung, and tumor neovascular blood vessels (Diebold et al., 2019). Loss of FECH activity was anti-proliferative for brain microvascular ECs, with no effect on macrovascular ECs (Basavarajappa et al., 2017). This was in contrast to reduced heme synthesis seen in macrovascular ECs as a result of aberrant serine synthesis (Vandekeere et al., 2018). The differential phenotypes of heme loss in microvasculature versus macrovasculature remain unclear and solicit further studies (Ghitescu and Robert, 2002; Sandoo et al., 2011).

Sprouting human umbilical vein ECs are highly glycolytic, producing up to 85% of ATP through the glycolysis pathway. During angiogenesis, endothelial tip and stalk cells dynamically switch their glycolytic activity depending on the energy demands of the tip cells and the proliferating stalk cells (De Bock et al., 2013). Recently, endothelial tip cells were reported to be less glycolytic during angiogenic cell differentiation (Yetkin-Arik et al., 2019), however more studies are warranted to validate such observations. Additionally, mitochondrial fatty acid oxidation has a role in proliferation of sprouting ECs (Schoors et al., 2015). While blocking heme production diminishes glycolytic capacity of retinal ECs (Shetty et al., 2020), it is as yet unclear whether heme regulation of EC metabolism varies between tip and non-tip ECs. Recent genomic analysis of murine choroidal ECs from neovascularization revealed potential metabolic candidates not found in healthy cells, suggesting targeting endothelial metabolism could be the way forward in vascular therapeutics (Rohlenova et al., 2020).

### Cytosolic Effects

Lack of heme synthesis also leads to incomplete formation of eNOS and reduced activity (Feng, 2012). Heme depletion *via* FECH inhibition led to decreased expression, hemylation, and activity of eNOS in retinal microvascular ECs (Basavarajappa et al., 2017). Heme inhibition by chemically blocking the second synthesis enzyme aminolevulinic acid dehydratase (ALAD) in rats led to reduced eNOS and downstream mediator soluble guanylyl cyclase (sGC), both important in maintaining regular cardiovascular function. These effects did not affect vascular tension and resulted in no change to arterial blood pressure (Bourque et al., 2010). But heme depletion-driven eNOS dysfunction led to impaired NO mediated vascular relaxation in bovine coronary arteries (Zhang et al., 2018). NO, a potent vasodilator, is pro-angiogenic and NO itself is known to inhibit hemylation of extramitochondrial *apo*-hemoproteins (Waheed et al., 2010).

It is important to note that heme overload in ECs also leads to abnormal angiogenesis. Silencing of the heme transporter FLVCR1a led to intracellular heme accumulation in microvascular ECs, but not in macrovascular ECs. This heme accumulation in microvascular ECs led to impaired angiogenesis, damaged vessel formation and embryonic lethality *in vivo* (Petrillo et al., 2018). Heme toxicity has been investigated previously in hemolytic diseases like sickle cell disease and thalassemia, where heme scavengers are helpful in reducing heme-induced ROS accumulation (Vinchi et al., 2013). In non-small cell lung cancer, tumor cells had elevated heme synthesis activity, increasing respiratory function of the ETC and enhancing tumorigenic properties like migration and invasiveness (Sohoni et al., 2019). This suggests in addition to heme loss being anti-angiogenic, heme synthesis overdrive can increase mitochondrial function, but this remains to be validated in ECs. It would be interesting to investigate whether heme mediates EC metabolism in neovascularized tumors in a similar fashion and whether heme synthesis blockers could be valuable as cancer therapies.

## Therapeutic Potential of Targeting Heme Synthesis in Neovascularization

Current therapeutic strategies targeting mitochondria involve key functions like mitochondrial division (Cassidy-Stone et al., 2008), ROS formation (Dhanasekaran et al., 2004), and metabolism (Mather et al., 2001; Csiszar et al., 2009) for age-related neurodegenerative diseases like Alzheimer’s, Parkinson’s, and Huntington’s (Lane et al., 2015). Meanwhile, anti-vascular endothelial growth factor (VEGF) therapies remain classic biologics used for neovascular diseases such as wet AMD, PDR, and multiple cancers (Jain, 2014). Until our and others’ work described above, there was no rationale for targeting heme synthesis as neovascularization therapy. But given the specific antiproliferative effects of FECH blockade in microvascular ECs, FECH inhibitors like *N*-methylprotoporphyrin have demonstrated potential in targeting neovascular pathologies, both *in vitro* and in the OIR mouse model (Basavarajappa et al., 2017; Pran Babu et al., 2020). Novel, drug-like FECH inhibitors are also a possibility (Corson et al., 2019; Sishtla et al., 2019).

Repurposing existing drugs for pathological angiogenesis also holds promise towards this end. Griseofulvin, an FDA-approved anti-fungal drug, has a long-known off-target effect of FECH inhibition (Brady and Lock, 1992; Liu et al., 2015). It has anti-angiogenic effects in retinal ECs, blocking proliferation, migration, and tube formation *in vitro* and reducing neovascularization *in vivo* comparable to intraocular anti-VEGF treatment, in both OIR and L-CNV mouse models (**Figure 1B**) (Basavarajappa et al., 2017; Pran Babu et al., 2020). Isoniazid, an anti-mycobacterial drug, decreases FECH expression while upregulating ALAS1 (Brewer et al., 2019), and thus could be tested for potential anti-angiogenic activity in neovascularization models. Other inhibitors of heme synthesis used *in vitro* include succinylacetone and salicylic acid that block ALAD and FECH respectively (Giger and Meyer, 1983; Gupta et al., 2013), however their use in preclinical angiogenesis models remains to be investigated.

Targeting mitochondrial proteins directly involved in ETC activity has limitations as well, with a direct consequence on mitochondrial function. However, extracellular supplementation of hemin (a more stable form of heme) is able to normalize some of the mitochondrial physiology, like eNOS levels, complex IV activity, and ETC function (Basavarajappa et al., 2017; Vandekeere et al., 2018; Shetty et al., 2020). Effect of FECH blockade can be titrated, with a dose dependent decrease in angiogenesis features observed in animal models and ECs in culture. Homozygous *Fech*^m1Pas^ mice have significantly reduced neovascular lesions, compared to heterozygous *Fech*^m1Pas^ mice. And heterozygotes themselves have reduced lesions compared to wild-type (Basavarajappa et al., 2017), suggesting a window of FECH antiangiogenic effects without toxicity. However, complete loss of *Fech* and *Alas1* are embryonically lethal to mice (Magness et al., 2002; Chiabrando et al., 2014a), highlighting the importance of modulating heme inhibition carefully.

Oral supplementation of heme, while still achieving therapeutic antiangiogenic effects of inhibitors, could be considered (Luan et al., 2017). In order to limit systemic toxicity, it would be helpful to localize therapeutic formulations to pathological tissue wherever possible. For example, in ocular neovascularization, therapeutic agents could be delivered through intravitreal or subretinal injection (Basavarajappa et al., 2017), or even as eyedrops if formulation allows; this is a promising area for future work. Therapeutic targeting specific to ECs could be included in drug delivery systems (Kawahara et al., 2013), since systemic deficiency in heme synthesis enzymes can lead to porphyrias. For example, erythropoietic protoporphyria is caused by toxic buildup of PPIX (Gouya et al., 1999). The phototoxic PPIX can be detrimental to cells, and is manipulated in photodynamic therapy (PDT) (Krammer and Plaetzer, 2008). However, it is unlikely that PPIX itself mediates anti-angiogenic effects, as ALA-PDT relies heavily on uptake of ALA (Wachowska et al., 2011). Moreover, as noted, hemin is able to rescue anti-angiogenic effects in ECs, even in the presence of PPIX build-up, suggesting that this mechanism is heme dependent and not due to PPIX toxicity.

## Conclusions and Future Prospects

Targeting intracellular heme, either *via* inhibition of synthesis through intermediary enzymes or blocking heme transport (through FLVCR) provides for a novel therapeutic strategy, one that is primed to be explored in detail in vascular biology. Key questions that need to be addressed are: Is the role of heme in angiogenesis limited to ETC and eNOS or do other heme-containing proteins aid in anti-angiogenic effects? Which enzymes in the heme synthesis pathway are the most effectively targetable for treating pathological angiogenesis? What are the key differences in microvascular and macrovascular heme synthesis, and can we manipulate these therapeutically? Proliferative ECs appear to be particularly sensitive to heme loss, but is this sensitivity only relevant in vascular tissues? Most importantly, we also need to elucidate the contribution of heme and heme pathway intermediates in maintaining normal endothelial cellular physiology, to devise better strategies for future therapeutic interventions.

## Author Contributions

TS, TC: wrote the paper, edited the paper, and approved final version.

## Funding

Related work in the Corson laboratory is supported by NIH/NEI R01EY025641, NIH/NCATS UL1TR001108, the Retina Research Foundation, the International Retinal Research Foundation, the BrightFocus Foundation, the Carl Marshall and Mildred Almen Reeves Foundation, and the Ralph and Grace Showalter Research Trust.

## Conflict of Interest

TC is a named inventor on patent applications related to this topic.

The remaining author declares that the research was conducted in the absence of any commercial or financial relationships that could be construed as a potential conflict of interest.
